# Diet and nutritional status among children 24–59 months by seasons in a mountainous area of Northern Vietnam in 2012

**DOI:** 10.3402/gha.v7.23121

**Published:** 2014-12-08

**Authors:** Le Thi Huong, Le Thi Thanh Xuan, Le Hong Phuong, Doan Thi Thu Huyen, Joacim Rocklöv

**Affiliations:** 1 Institute for Preventive Medicine and Public Health, Hanoi Medical University, Hanoi, Vietnam; 2 Epidemiology and Global Health, Department of Public Health and Clinical Medicine, Umeå University, Umeå, Sweden

**Keywords:** Vietnam, nutritional status, diet, children, Tuyen Quang, seasons

## Abstract

**Background:**

Seasonal variation affects food availability. However, it is not clear if it affects dietary intake and nutritional status of children in Vietnam.

**Objectives:**

This paper aims at examining the seasonal variation in nutrition status and dietary intake of children aged 24–59 months.

**Design:**

A repeated cross-sectional study design was used to collect data of changes in nutritional status and diets of children from 24 to 59 months through four seasons in Chiem Hoa district, Tuyen Quang province, a predominately rural mountainous province of northern Vietnam. The quantitative component includes anthropometric measurements, 24 hours dietary recall and socio-economic characteristics. The qualitative component was conducted through focus group discussions (FGDs) with mothers of the children surveyed in the quantitative component. The purpose of FGDs was to explore the food habits of children during the different seasons and the behaviours of their mothers in relation to the food that they provide during these seasons.

**Results:**

The prevalence of underweight among children aged 24–59 months is estimated at around 20–25%; it peaked in summer (24.9%) and reached a low in winter (21.3%). The prevalence of stunting was highest in summer (29.8%) and lowest in winter (22.2%). The prevalence of wasting in children was higher in spring and autumn (14.3%) and lower in summer (9.3%). Energy intake of children was highest in the autumn (1259.3 kcal) and lowest in the summer (996.9 kcal). Most of the energy and the nutrient intakes during the four seasons did not meet the Vietnamese National Institute of Nutrition recommendation.

**Conclusions:**

Our study describes some seasonal variation in nutrition status and energy intake among children in a mountainous area northern Vietnam. Our study indicated that the prevalence of stunting and underweight was higher in summer and autumn, while the prevalence of wasting was higher in spring and autumn. Energy intake did not always meet national recommendations, especially in summer.

Nutrition is very important for the growth and development of children (1). Child development depends on many factors such as: genetic, endocrine, autonomic nervous system and nutritional status (1). The first three factors certainly play important roles for cognitive development of children, while proper nutrition supplies necessary substances for maximisation of the potential development (1).

Results from National Nutrition Surveillance, from 2000 to 2009, showed that malnutrition among children aged less than 5 years in Vietnam (especially severe malnutrition and underweight) has reduced significantly and sustainably. However, the prevalence of underweight among children aged less than 5 years in some mountainous areas was up to 25–35% and stunting prevalence up to 37–47% (2–4). While in the urban areas, the prevalence of underweight was around 10% and prevalence of stunting was around 20% (5).

Xuan Quang is an agriculture area where subsistence farming is practiced. It is assumed that differences of weather through seasons may have an impact on household food availability and food consumption, which affects the child's nutritional status (6–9). Few studies have examined the effect of different season on nutrition status of children (10–13). The aims of this study are: 1) to describe the seasonal change of children's diet in Xuan Quang commune, Chiem Hoa district, Tuyen Quang province; 2) to describe the seasonal change of nutrition status of children from 24 to 59 months in Xuan Quang commune; 3) to explore the food habits of children and behaviours of the mothers in relation to the food that they provide to their children during these different seasons. This study would help develop strategies to protect vulnerable populations to overcome the potential negative effects of seasonal variability in climate.

## Methods

### Study setting

Xuan Quang is located in a rural mountainous area in the North of Chiem Hoa district where the climate varies by season. The commune has 13 villages with 10 groups of ethnic minority people (78.3%), while the Kinh group contains 11.7% of the population. The number of households in Xuan Quang was 1,192, of which 234 and 331 households were poor and near to poor, respectively (following the ranking of the local authority). Based on the commune health centre report in 2011, the commune had 5,000 people, of them about 200 children aged 24–59 months (14).

**Fig. 1:**
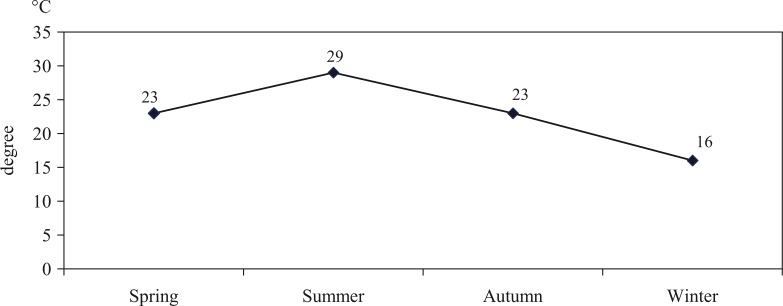
Average temperature of Tuyen Quang province in 2012 (15).

There are four seasons in Tuyen Quang province: spring, summer, autumn and winter. The dry and cold winter lasts from November to February. The spring lasts from March to April; rainy hot summer lasts from May to August. Autumn lasts from September to October. Autumn and spring are the two short transitional seasons. The annual rainfall average of the region is normally in the interval of 1,295–2,266 mm; the average temperature varies from 26 to 29°C (Fig. 1), and the annual humidity average is around 85%. Resulting from geographic location, Tuyen Quang province is divided into two regions with different weather conditions; the northern part of this province, including Xuan Quang commune, where this study is carried out, has a longer winter, lower temperature and much more rainfall in summer (15).

### Study subjects

#### Quantitative study

All children 24–59 months living in Xuan Quang commune of Chiem Hoa district without congenital, chronic or acute diseases were included in each survey together with their mothers. We selected this group because between the ages of 2–5, children start eating with their family and are less likely to receive special diets.

#### Qualitative study

We invited mothers who have children from 24 to 59 months (6–8 mothers for each focus group discussion).

Mothers/caregivers were informed about the purposes of the survey, and asked to sign an informed consent before participating in the study.

### Study design and data collection

A repeated cross-sectional study design was used to collect data of seasonal changes in nutrition status and nutrients intake of children from 24 to 59 months. Data were collected at the beginning of spring (March), summer (June), autumn (September) and winter (December) (15).

A list of mothers and children was provided by village health workers. The mothers and children were invited by the village health workers to the kindergarten, village meeting room or community health centre for the anthropometric measurements and the 24-hour recall interviews. The mothers that participated in the FGDs were invited to a separate room for these sessions.

#### Anthropometric measurement

All children aged 24–59 months were measured for weight and height by project researchers with support from village health workers. We used Microtoise height meter for measuring children's height with the precision of 0.1 cm. Weight was measured by electronic Nhon Hoa scales with the precision of 100 g. All children were undressed for weighing. In each survey, the well-trained staff from the Nutrition Department at Hanoi Medical University conducted one measurement for each child.

#### Nutrition status identification

Three indicators were used to identify the nutritional status of the children, using the WHO recommendation applied in Vietnam since 2006: weight for age, height for age and weight for height (16–18). Underweight was defined as: weight for age z-score (WAZ) ≤2; stunting was defined as: height for age z-score (HAZ) ≤2; and wasting was defined as: weight for height z-score (WHZ) ≤2 (19). All children of the commune aged 24–59 months were included in the anthropometric measurement at each study time (spring: 195 children; summer: 237 children; autumn: 196 children and winter: 225 children).

#### Food intake of children (24 hours recall)

To be able to assure a large enough sample size to perform valid statistical tests with a power of 80% and a significance level of 5%, we performed sample size calculations with a standard *t*-test. The sample size calculations were subject to the outcome of food intake of children:

Sample size was calculated based on the following formula (20):

$$N=\frac{t^{2}.\delta^{\text{2}}.n}{e^{2}.n+t^{2}.\delta^{\text{2}}}$$

where:

*N*: Sample size (people)

*t*: The standard percentile (=2 with a probability of 0.954)

δ: Standard Deviation (base on previous study estimated to 400 kcal)

*n*: Total number of children from 24 to 59 months of population

*e*: Relative precision (70 kcal was selected)

The sample size was calculated to include 79 children. An additional 10% was added to account for children who may not completed the survey. This resulted in a sample of 90 children for 24-hour recall in each survey.

Based on the list of children aged 24–59 months of the commune that was provided by the commune health staff, 90 children and their mothers were then randomly selected for each season. To make the balance for three age groups: 24–35 months, 36–47 months and 48–59 months, we randomly selected 30 children from each age group.

The 24-hour recall questionnaire was designed to collect data in the four seasons. One day 24-hour recall was collected in normal day for each season. The face-to-face interview was conducted by the well-trained staff from Nutrition Department at Hanoi Medical University. The researchers were equipped with instruments like food pictures, various kinds of spoons and mugs to help the mothers estimate the amount of food provided to children during the day. In this study area, the mothers were the main caregivers of children, who prepared foods and fed the children. In case a mother could not feed the children during the day, their grandfather or grandmother usually fed the children using food prepared by the mother.

#### Focus group discussion

Two focus group discussions (FGDs) (6–8 mothers) were applied during the first round of this study. The main focus of the FGDs was to explore the mother's view on their children's food habits depending on season. This discussion also focused on exploring the mother's behaviours regarding food selection and preparation for children through these seasons. The FGD was conducted by researchers with experience in qualitative methods to ensure quality of the data (21).

Mothers/caregivers were randomly selected and invited to a community health centre for the FGDs. The FGD was conducted in a separate and private room to make mothers and researchers feel comfortable. The guideline was developed to facilitate the FGDs and to create a positive interview atmosphere, where the informants could share the experiences. Each focus group discussion lasted for about 45 min. It was facilitated by one researcher with the support from another researcher responsible for taking notes.

### Data analysis

Anthropometric data was entered in Anthro software version 2005 and analysed using STATA version 10.0 (22). Data of 24-hour recalls were entered and analysed in Access office package for nutrition analyses. A module in Access had been developed by Vietnam National Institute of Nutrition (NIN).

The ranksum-test was used to test for significant difference between the medians of two groups and Chi-square test was used to test for significant differences between the proportions of groups. A *p*-value under 0.05 was considered statistically significant. Kruskal-Wallis test was used to test for significant difference in the medians of more than two groups.

Qualitative data were analysed by using a content analysis method. The information was coded following themes, and the analysis was carried out by the researcher conducting the FGD. More specifically, the researcher read the notes, arranged the content following the topic, coded, analysed into themes, and presented integrated with quantitative data (23).

### Ethical considerations

The protocol of this study was approved by the Scientific and Ethical Committee in Biomedical Research, Hanoi Medical University. All subjects in the study were asked for their informed consent before collecting data, and all had complete rights to withdraw from the study at any time without any threats or disadvantages.

## Results

The number of children involved in the anthropometric measurement during the spring, summer, autumn and winter were 195, 237, 196 and 225, respectively.

This study did not show a large difference in mean weight and height of children between seasons. The mean weight varied from 12.4 to 12.7 kg and the mean the height varied from 92.4 to 93.1 cm. There was no significant difference between the seasons on the mean WAZ, HAZ and WHZ of the children (Table 1).

**Table 1:** Mean weight, height and z-score of the children following the seasons

	Mean	SD	*p*
Weight			
Spring	12.7	2,1	*p*>0.05
Summer	12.6	2,0	
Autumn	12.7	2,2	
Winter	12.4	1,9	
Height			
Spring	93.1	8.1	*p*>0.05
Summer	93.0	7.8	
Autumn	92.6	8.0	
Winter	92.4	8.3	
WAZ (weight for age z-score)			
Spring	−1.4	0.9	*p*>0.05
Summer	−1.4	0.9	
Autumn	−1.4	1.0	
Winter	−1.4	0.9	
HAZ (height for age z-score)			
Spring	−1.5	1.2	*p*>0.05
Summer	−1.5	1.2	
Autumn	−1.5	1.2	
Winter	−1.5	1.2	
WHZ (weight for height z-score)			
Spring	−0.8	1.1	*p*>0.05
Summer	−0.8	1.1	
Autumn	−0.8	1.2	
Winter	−0.9	1.1	

**Fig. 2:**
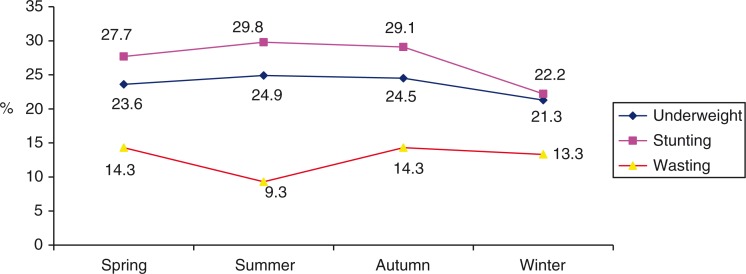
Prevalence of Underweight, Stunting and Wasting by season.

Figure 2 presents the seasonal changes in the prevalence of children's malnutrition. The prevalence of stunting was around 20–30%, highest in summer (29.8%) and lowest in winter (22.2%). There was no significant difference on stunting prevalence between age groups (24–35 months, 36–47 months and 48–59 months). The proportion of underweight was lowest in winter (21.3%) and ranging from 23 to 24% in the other seasons with no statistical significant difference (Chi-squared test, *p*>0.05). By contrast, the proportion of wasting children was higher in spring and autumn (14.3%) and lower in summer (9.3%). There was no statistical significant difference among seasons (*p*>0.05).

The results of the focus group discussion indicated that the main food source supplying energy intake for children in this commune was rice. Foods rich in protein available to access for local people in all seasons were pork, fish, eggs and bean. Crabs, eels, snails and mussels are available mainly in the summer. However, some nutritious foods were not fed to children because mothers did not know how to prepare these food items or they were afraid that their children risk having gastrointestinal problems after eating these.

My neighbour *don't know how to cook crabs, she doesn't know what the nutrient value of crabs* are. Many mothers are afraid that when their child *eat crab, they may have* gastrointestinal problems. (Mother, 28 years old, Xuan Quang. Mentioned during the FGD)

The data from FGD also indicated seasonal variation in the availability of vegetable and fruit, but not for protein-rich foods. During winter, more green leaf vegetables are being consumed compared to other seasons.

Table 2 showed a significant seasonal variation in total energy intake (*p*<0.01): highest in autumn (1259.3 kcal), lower in spring and winter and lowest in summer (996.9 kcal). In addition, the amount intake of carbohydrates protein and lipids among children in autumn were higher than other seasons (*p*<0.05). Most of the children's diets did not meet the recommendation of the NIN for energy, protein, carbohydrates and lipid intake.

**Table 2:** Energy, protein, glucid and lipid intake of children aged 24–59 months by season

Age group (*n*=90)	Recommendation	Spring (*n*=90)	Summer (*n*=90)	Autumn (*n*=90)	Winter (*n*=90)	(Kruskal-Wallis test)
Energy (kcal/day)						
24–35 months	*1,180*	1041.3	991.7	1194.2	1008.7	>0.05
36–47 months	*1,180*	1049.6	957.9	1247.3	1166.1	**<0.05**
48–59 months	*1,470*	1103.7	1053.9	1327.7	1053.6	**<0.05**
All		1067.8	996.9	1259.3	1075.4	**<0.01**
Protein (g/day)						
24–35 months	*35–44*	39.3	40.7	48.2	40.2	>0.05
36–47 months	*35–44*	43.9	40.2	51.5	42.8	>0.05
48–59 months	*44–55*	40.7	44.7	54.4	38.3	**<0.01**
All		41.1	41.4	51.5	40.4	**<0.01**
Lipid (%=lipid energy demand/total energy)						
24–35 months	*44–51*	18.4	23.5	28.1	24.3	**<0.05**
36–47 months	*44–51*	23.6	19.0	29.5	24.4	>0.05
48–59 months	*32–40*	20.1	22.5	30.6	20.1	**<0.05**
All		20.5	22.2	29.5	22.8	**<0.01**
Glucid (g/day)						
24–35 months	*175–202*	178.3	153.4	187.3	157.8	**<0.05**
36–47 months	*175–202*	164.2	157.0	192.9	194.4	**<0.05**
48–59 months	*219–251*	189.4	166.6	206.9	180.5	**<0.05**
All		178.8	157.0	196.1	177.7	**<0.01**

FGD with mothers in the local study area showed a similar result with mothers indicating that children eat more in the wintertime:

It also depends on the weather condition, for example, in autumn the children often eat better whereas in hot conditions of summer children prefer to eat fruits rather than main diets. (Mother, 28 years old, Xuan Quang. Mentioned during the FGD)

**Fig. 3:**
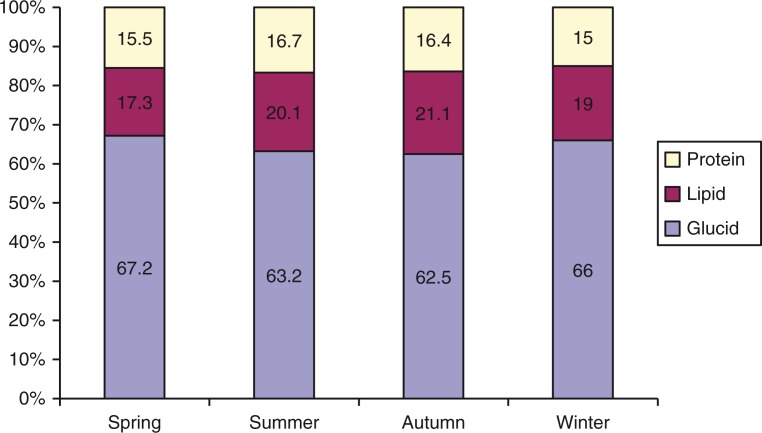
Proportion of substances supplying total energy in diets of children aged 24–59 months by season.

Figure 3 presents the proportion of total energy intake in diets of children by season. The results show that glucid is the main macronutrient for energy supplement, and represented more than 60% in all seasons, highest in spring (67.2%) and in winter (66%).

*In the summer*, the *kids will eat more junk food*, the number of *main meals is still the same but the child eats less*. *Mainly because of the weather*. The *hot* weather makes children *tired and as a consequence they* do not eat much. (Mother, 28 years, Xuan Quang. Mentioned during the FGD)

Energy supplement from protein was 14%, which was lower in spring and winter. There was no statistical significant difference of supplement balance among protein (P), glucid (G) and lipid (L) between seasons (*p*>0.05). In general, the proportion of the energy intake from nutrients intake (P:L:G) is similar to the recommendation (P:L:G=14:20:66).

Findings from group discussion supported that children often eat more protein-rich foods in the autumn:

In the autumn, they eat more than the pre-harvest period. There is no fasting in pre-harvest period, mother do not want to buy meat or fish because they do not have money. (Mother, 28 years old, Xuan Quang. Mentioned during the FGD)

A difficulty identified through the FGD was that mothers find it harder to feed their children with protein-rich food because of inaccessibility, particularly, in rainy season when it can be very difficult to get to the market. Another reason that many mothers mentioned is the poor economic condition, and the lack of money to buy good foods such as beef, milk, etc.

Table 3 presents seasonal intakes of some vitamins, minerals and micronutrients by age group of children. The amount of vitamin A intake for children followed the recommendation whereas the amount of iron and calcium are below the recommendations. Results also show that the intake of iron and calcium are higher in the summer and the autumn (*p*<0.05). The amount of phosphorus consumed met the requirement; the resulting Ca/P ratio is rather high with 1:1.2 as the lowest level.

**Table 3:** Actual amount of vitamin and mineral intake by season and children age group

Age group (*n*=90)	Recommendation	Spring (*n*=90)	Summer (*n*=90)	Autumn (*n*=90)	Winter (*n*=90)	*p* (Kruskal-Wallis test)
Vitamin A (mcg/day)						
24–35 months	*400*	558.7	716.2	843.8	459.7	**<0.05**
36–47 months	*400*	519.1	687.4	780.3	486.5	>0.05
48–59 months	*450*	544.6	802.5	795.9	463.0	**<0.01**
All		542.7	769.9	**805.6**	469.5	**<0.01**
Calci (mg/day)						
24–35 months	*500*	346.2	537.0	433.4	394.8	>0.05
36–47 months	*500*	316.9	376.2	491.8	308.3	>0.05
48–59 months	*600*	300.2	381.0	403.7	269.8	**<0.01**
All		320.5	**466.6**	444.3	322.5	**<0.01**
Iron (mg/day)						
24–35 months	*7.7*	6.1	6.6	10.8	4.9	**<0.01**
36–47 months	*7.7*	5.5	6.4	6.9	6.9	**<0.05**
48–59 months	*8.4*	6.5	8.3	10.7	6.2	**<0.05**
All		6.1	6.9	**8.4**	6.0	**<0.01**
Ca/P						
Proportion Ca/P	*1.0:1.5*	1.0:1.7	1.0:1.2	1.0:1.5	1.0:1.7	

Table 4 indicated that malnourished children generally had a lower intake of glucid in spring and lower intake of lipid in summer (*p*<0.05). The table also shows that underweight children usually had a lower intake of energy, protein, carbohydrate and lipid than normal-weight children (*p*>0.05).

**Table 4:** Nutritional status (underweight) and dietary intake of children by season

	Spring (*n*=90)		Summer (*n*=90)		Autumn (*n*=90)		Winter (*n*=90)	
	
	Malnutrition	Non-malnutrition	Malnutrition	Non-malnutrition	Malnutrition	Non-malnutrition	Malnutrition	Non-malnutrition
Energy intake	981.4	1092.5	964	1008.2	1162.4	1290.7	1199.9	1050.5
*p* (ranksum-test)	>0.05		>0.05		>0.05		>0.05	
Protein	40.1	41.4	40.4	41.8	48.4	52.5	42.9	39.9
*p* (ranksum-test)	>0.05		>0.05		>0.05		>0.05	
Glucid	150.4	187.0	156.4	157.2	179.8	201.4	188.7	175.5
*p* (ranksum-test)	**<0.05**		>0.05		>0.05		>0.05	
Lipid	23.0	19.8	18.4	23.6	26.9	30.3	30.7	21.2
*p* (ranksum-test)	>0.05		**<0.05**		>0.05		**<0.05**	

## Discussion

The prevalence of underweight was highest in the summer (24.9%) and lowest in winter (21.3%). This level is much lower than the findings of another study in rural and mountainous areas carried out in Quan Son of Thanh Hoa province (about 30%) (24). The results showed that the proportion of stunting in Xuan Quang was highest in summer (29.8%), followed by autumn (29.1%), spring (27.7%) and winter (22.2%). The stunting rate, however, reflects the malnutrition status of children over a long time, while this study was carried out in 1 year. Therefore, the above results could not show the correlation between stunting prevalence and climatic extreme events and climate change. For such studies, it would be necessary to carry out longitudinal studies to examine the correlation between children malnutrition status and climate change over many years. However, this study may still be used as a reference if shifts in the seasonality of climatic extreme events would occur that would potentially effect the populations. Prevalence of stunting in our study was lower than that in a mountainous district of Thanh Hoa province (about 40%) (25). Our study found that wasting (the indicator that better reflects current acute malnutrition) prevalence ranged from 9.3 to 14.3%. Unlike underweight and stunting, the prevalence of wasting was highest in spring and autumn (14.3%), and lowest in summer (9.3%). This number was higher than the reported statistics of developing countries with 9% (26). A study of L. Loutan and J-M. Lamotte in Niger showed that a reduction of weight of children under 5 years from February to May caused a rapid increase of wasting from 7% in November to 17% in May (the peak time of the dry season is from February to May in Nigeria) (11).

The result from our study showed that the prevalence of stunting and underweight was higher in summer time in comparison with other seasons. A significant difference was, however, only observed for wasting in the summer time. In the study from Nigeria, children's weight did not increase in dry season but reduced by 113 g. After the dry season the children's weight increased again (11). Our study's results could not reflect the acute malnutrition of children caused by lower energy intake in summer due to time limitations of our study. The anthropometric data were collected at the beginning of the four seasons, so that the low prevalence of the wasting (acute malnutrition) at the beginning of summer (9.3%) can be explained by better food intake during the spring time, then the higher prevalence of wasting at the beginning of autumn (14.3%) as a result of poor nutrition intake during summer time. Moreover, the nutritional status of children not only depends on the food intake but also on others factors such as infectious illness, children caring, among other factors.

Although there was some significant seasonal variation in Xuan Quang commune in nutritional status, seasonality did, in general, not affect the production and access to food. Diets of the children were adapted to the circumstances, and mothers tried to get enough food per each four food-type group in each meal of the children. It is likely due to this adaptation that we observed that the prevalence of underweight and malnutrition did not differ by seasons. In comparison with Quan Son district of Thanh Hoa province, where a similar pattern was observed, this study area had poorer economic conditions and more seasonally contrasting weather patterns (24).

There was a significant seasonal difference in nutrition intake of children aged 24–59 months (*p*<0.01). The average energy intake, amount of proteins, lipid, glucid intake of children were all highest in autumn (*p*<0.01). The results from qualitative section explained that cool weather in autumn made children eating more than other seasons. Nutrition intake of children in all four seasons mostly failed to reach the recommendation for energy and nutrients. This result was similar with the result of NIN studies showing poor density of energy intake, fatty food, animal fatty and micronutrients (27). This finding resulted from the mothers work burden, especially in rural and mountainous areas, where they have less time to feed their children directly. In addition, results from group discussions showed the lack of mother's knowledge on caring and requirements of children's nutrition:

It's because of mothers’ knowledge. For example, they do not know how to prepare food from crabs as well as nutrients form crabs. Some children do not eat food from crabs, some have digestion problems. (Mother, 27 years old, Xuan Quang. Mentioned during a FGD)

The structure of the energy intake among children aged 24–59 months was similar in the four seasons. Glucid was the main substance supplying energy in all seasons with more than 60% (highest in spring and winter with 67 and 66%, respectively), which exceeded the recommendation (66%). Energy supplement from protein was higher than the recommendation's balance, ranging from 15 to 16.7%. This is consistent with the study by Le Thi Hop et al. (15.4%), which also showed an improvement in children's diets (28).

Iron is also an important and essential micronutrient for the human body, especially for young children. The amount of iron intake of children in our study did not meet the recommendation. Significantly higher amounts of iron intake were found in autumn and summer than spring and winter. Underweight children usually had lower energy intake, protein, glucid and lipid than children who were not underweight, where no significant difference was found. This result was different from another study finding that malnourished children had lower energy consumption, lower energy intake from animal protein and lipids than normal children (29); therefore, the higher prevalence of malnutrition may combine with the high prevalence of iron deficiency.

Our study found no differences in energy intake between stunting, wasting children and normal children in all four seasons (*p*>0.05). The sample size of this study may, however, not have been big enough to show a statistical significant difference for the differences observed. It is necessary to conduct a longer-term prospective study to find out the correlation between children's diets and the prevalence of malnutrition in relation to climate variability in the future.

## Conclusion

Our study shows some seasonal variation in nutrition status and energy intake. Prevalence of stunting and underweight were observed to be higher in summer and autumn, while the prevalence of wasting was observed to be higher in spring and autumn. Energy intakes did not always meet the recommendation, especially in summer.
